# Supplementary material for: New versions of maps and connected spaces via supra soft sd-operators
Source: PLoS One. 2024 Oct 24;19(10):e0304042. doi: 10.1371/journal.pone.0304042 (PMC11500933; doi:10.1371/journal.pone.0304042)
Supplement: Attachment — Submitted filename: Response to Reviewers--PONE-D-24-09504.pdf [file pone.0304042.s001.pdf]

Response to Reviewers

Manuscript ID: PONE-D-24-09504
Title: New versions of maps and connected spaces via supra soft sd-operators

Reply to Reviewer 1:

First of all, we would like to thank you for taking the time and effort necessary to review the manuscript. We sincerely appreciate all valuable comments and suggestions, which helped us to improve the quality of the manuscript.

Secondely, we agree with you. The English language and style were needed to be revised. So, we revised the spelling and grammar errors via professional scientific editing service from Department of Languages and Translation / College of Sciences and Arts / Northern Border University, which is being highlighted in the paper. [In special , See Abstract, Introduction, and Conclusion sections. Also, in the introduction for the main sections].

Moreover, we improved the manuscript according to your mentioned points as follows:

1. In page 2, we corrected "In addition, we use..." instead of "In addition, we used ..."
2. In page 6, we corrected "Let (G, Δ) be both..." instead of "Let (G, Δ) is both ..."
3. We improved the way of presenting literature review by focusing on the mentioned topics in the Introduction section. [See Introduction section]
4. We revised the proof (2) ⇒ (1) of Theorem 3.7.
5. We unified the abbreviation for "supra soft" by "SS-" for all the paper.
6. In Example 4.21, the typo of (X_4, Δ), is corrected.
7. We investigated the importance of studying topological concepts in supra topologies, in special rough sets models.
8. We wrote a brief introduction for the main sections to improve the way of presentation.
9. [You are right] We linked the obtained results with their counterparts in classical settings, specifically in Remark 4.20 and Examples 4.21.

10. The conclusion section is improved by mention some future directions and demonstrating the importance of writing the manuscript.
11. Depending of the above arguments, the References list is updated.[see the References]

Reply to Reviewer 2:

First of all, we would like to thank you for taking the time and effort necessary to review the manuscript. We sincerely appreciate all valuable comments and suggestions, which helped us to improve the quality of the manuscript.

Secondely, we agree with you. The paper was needed to be revised. So that, we improved the manuscript according to your mentioned notes as follows:

1. We provided the main contributions of this paper in Abstract, Introduction, and Conclusion sections. Also, in the introduction for the main sections].
2. We merged paragraphs 3, 4, and 5 into one paragraph.
3. We unified the abbreviation for “supra soft” by “SS-” for all the paper.
4. In page 5, we replaced “(G^c, Δ) is proper supra closed soft subset” by “(G^c, Δ) is proper SS-closed subset”
5. We corrected the typo in Remark 3.8.
6. [You are right] We linked the obtained results with their counterparts in classical settings, specifically in Remark 4.20 and Examples 4.21.
7. We investigated the importance of studying topological concepts in supra topologies and infra topologies in a separated paragraph in the introduction section and focusing on the mentioned topics. [paragraph 1]
